# Clinical impact of myosteatosis measured by magnetic resonance imaging on long-term outcomes of hepatocellular carcinoma after radical hepatectomy

**DOI:** 10.1186/s12893-023-02188-z

**Published:** 2023-09-15

**Authors:** Kozo Yoshikawa, Mitsuo Shimada, Yuji Morine, Tetsuya Ikemoto, Yu Saito, Shinichiro Yamada, Hiroki Teraoku, Shoichiro Takao

**Affiliations:** 1 The Department of Surgery, The University of Tokushima, 770-8503 Kuramoto-cho Tokushima, Japan; 2 The Department of Diagnostic Radiology, Graduate School of Health Sciences, The University of Tokushima, 770-8503 Kuramoto-cho Tokushima, Japan

**Keywords:** Myosteatosis, Hepatocellular carcinoma, Magnetic resonance imaging, Radical hepatectomy, cancer-specific survival

## Abstract

**Aims:**

A variety of factors have been reported to affect long-term outcomes after radical resection of hepatocellular carcinoma (HCC). However, the indicators remain controversial. The purpose of this study was to evaluate the relationship between myosteatosis of the multifidus muscle and long-term outcomes after radical surgery for HCC.

**Methods:**

We retrospectively analyzed clinicopathological data for 187 patients with HCC who underwent radical surgery at Tokushima University between January 2009 and December 2020 and measured the density of fat in the multifidus muscle at L3 on their preoperative magnetic resonance images (MRI). Associations of myosteatosis and clinicopathological factors with long-term outcomes were evaluated.

**Results:**

The patients were divided into a myosteatosis-negative group (n = 122) and a myosteatosis-positive group (n = 65). The cancer-specific survival rate after hepatectomy was significantly worse in the myosteatosis-positive group than in the myosteatosis-negative group (p = 0.03). Univariate analysis identified multiple tumors, stage III/IV disease, an alfa-fetoprotein level ≥ 10 ng/ml, PIVKA-II ≥ 400 AU/ml, vp(+) status, and myosteatosis to be prognostic factors for cancer-specific survival. Multivariate analysis revealed multiple tumors, an alfa-fetoprotein level ≥ 10 ng/ml, and myosteatosis to be independent prognostic factors.

**Conclusions:**

Myosteatosis measured by MRI is a simple and useful predictor of the long-term outcome after radical surgery for HCC.

## Introduction

Radical surgery offers the possibility of a cure in patients with resectable hepatocellular carcinoma (HCC), and a variety of factors that affect long-term outcomes have been reported. Compared with the muscle mass, skeletal muscle is a good indicator of muscle quality and muscle strength in a healthy aging population [1]. Hence, low attenuation of skeletal muscle may potentially be more reflective of preoperative physical fitness and the capacity to recover from aggressive treatment. the clinical and biological variation of muscle phenotypes in reduced muscle attenuation suggest that myosteatosis is determined by increased intramyocellular infiltration and may be more predictive of poorer survival than muscle mass alone [2, 3].

The definition of the myosteatosis is alteration in the muscle quality due to the increased proportion of lipid accumulation in the muscle [4]. And this mean the loss of skeletal muscle mass and a pathological change, which is caused mainly by intramuscular infiltration of adipose tissue that is highly correlated with metabolic abnormalities and decreased physical strength and mobility [5]. The extent of infiltration worsens with progression of cancer and systemic inflammation and could be related to cancer cachexia, a syndrome of severe weight loss and muscle wasting. Correlation with cancer cachexia might explain why myosteatosis has such a strong impact on survival [3]. Muscle depletion and changes in muscle tissue composition occur with age and cancer-induced malnutrition. Muscle depletion is characterized by a gradual synergistic decline in skeletal muscle mass and high fat infiltration in muscle tissue [6].

Myosteatosis is an indicator of lower muscle quality whereas sarcopenia is considered a phenotype of decreased muscle quantity. Myosteatosis was found to be associated with shortened survival in patients with esophagogastric cancer; however, the prognostic impact of myosteatosis on mortality in patients with HCC was unclear [7–10]. There is increasing evidence to suggest that sarcopenia is correlated with the prognosis of cancer but evidence of the impact of myosteatosis on cancer outcomes are scarce.

Several methods for measurement of myosteatosis have been attempted in the research field, including a body imaging technique, bioimpedance analysis, and anthropometric measures. Myosteatosis is usually diagnosed by computed tomography (CT) because skeletal muscle volume can be measured at the same time as the mean radiodensity of skeletal muscle [11, 12]. However, magnetic resonance imaging (MRI) offers higher contrast in soft tissue and could be good modality compared with CT for visualizing skeletal muscle [3, 13, 14]. A recent report described a novel method for assessment of myosteatosis without invasion in various types of cancer in which MRI is used to determine the signal intensity (SI) of skeletal muscle [15].

We hypothesized that muscle attenuation would be predictive of key surgical outcomes following resection of HCC and became interested in determining whether the presence of myosteatosis preoperatively can predict complications and be associated with disease-free and cancer-specific survival or overall survival. The purpose of this study was to evaluate the relationship between myosteatosis and long-term outcomes after surgery for HCC.

## Methods

One hundred and eighty-seven patients with HCC who underwent curative hepatectomy in the Department of Surgery at Tokushima University between January 2009 and December 2020 were retrospectively investigated. All patients were performed CT and MRI routinely. Information on the perioperative characteristics of patients who underwent primary resection of the liver, including patient age, sex, body mass index, comorbidities, blood test results, operation time, blood loss, and postoperative complications, were obtained from hospital records and operation notes. Clinical staging was determined preoperatively by ultrasonography and thoracoabdominal CT and MRI. Pathological findings were defined in accordance with pathological and morphological parameters, and the Japanese Tumor–Node–Metastasis stage was determined in accordance with the criteria outlined by the Liver Cancer Study Group of Japan [16]. Information on dates and causes of death was collected from follow-up data based on clinical examinations performed every 3–6 months during the 5 years after surgery. This study aimed to evaluate the relationship between the pre-operative myosteatosis and survival after hepatectomy.

### Definition of postoperative complications

The severity of postoperative complications was evaluated using the Clavien–Dindo classification system. Postoperative complications were defined as conditions classified as Clavien–Dindo grade IIIa or higher [17].

### Acquisition of MRI scans

MRI of the upper abdomen was performed in all cases for detection and/or follow-up of a hepatic tumor. All MRI scans were obtained using a 1.5-Tesla scanner (Signa Explore, GE Medical Systems, Milwaukee, WI, USA) with a 12-channel body coil. For each subject, the MRI protocol included an axial non-contrast T1-weighted gradient echo sequence with dual echoes (in-phase and out-of-phase) under a single breath hold. The MRI acquisition parameters for the T1-weighted gradient echo sequence were as follows: method, gradient echo; mode, 2D; repetition time/echo time, 150/2.3 (out-of-phase), 4.9 (in-phase) msec; flip angle, 80°; field of view, 360 × 360 mm, image matrix size, 300 × 192; slice thickness slice gap, 7/2 mm; number of excitations, 1; and acquisition time, 16 s.

### Measurement of fat fraction of multifidus muscle

In this study, we refer the previous report to calculate the myosteatosis [18, 19].

DICOM (Digital Imaging and Communication in Medicine) data for each subject’s MRI scans were sent to a commercially available workstation (Synapse Vincent Vx; Fujifilm Healthcare, Lexington, MA, USA).

**Fig. 1:**
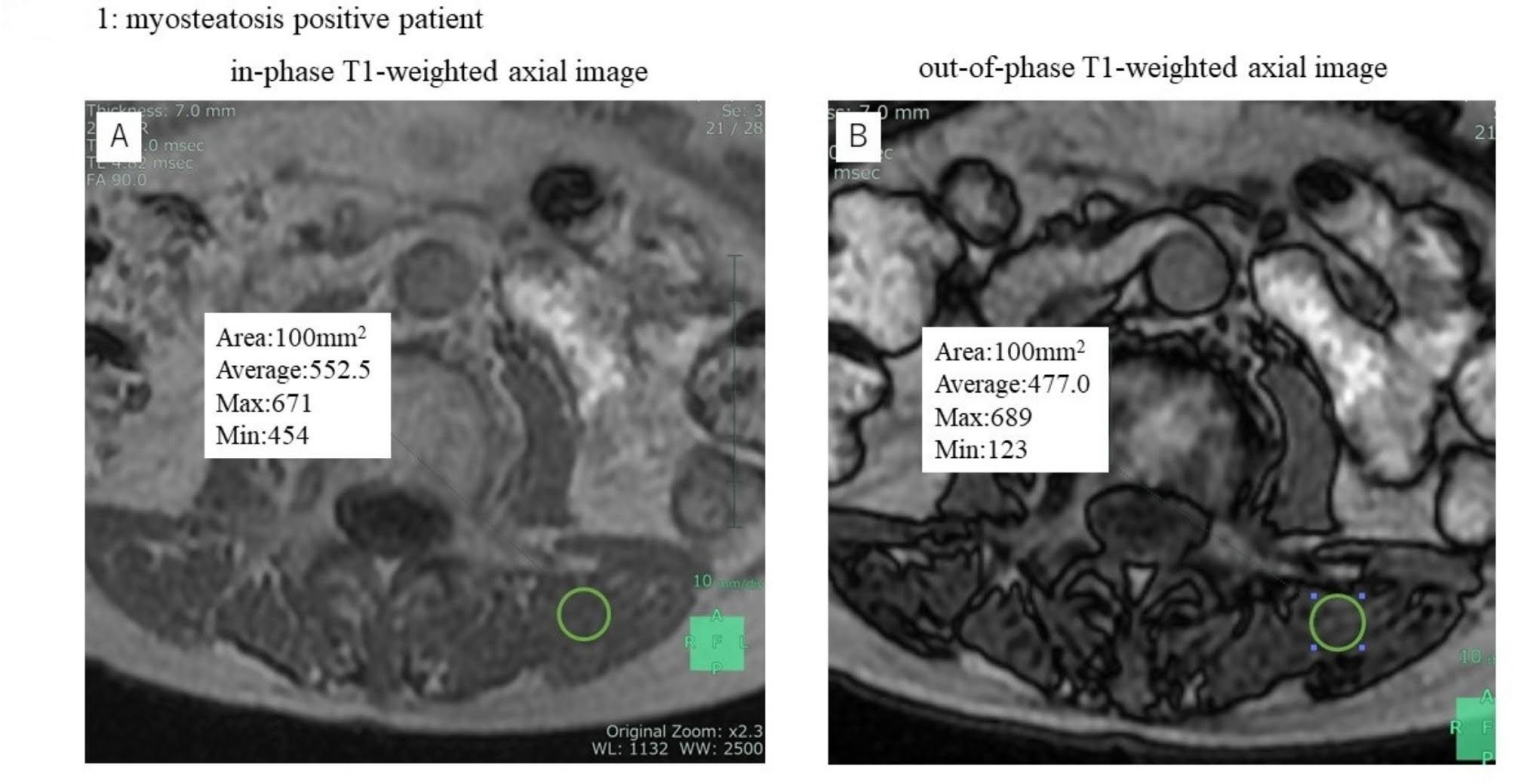
The representative MRI findings in myosteatosis positive patient in-phase T1-weighted axial image out-of-phase T1-weighted axial image

**Fig. 2:**
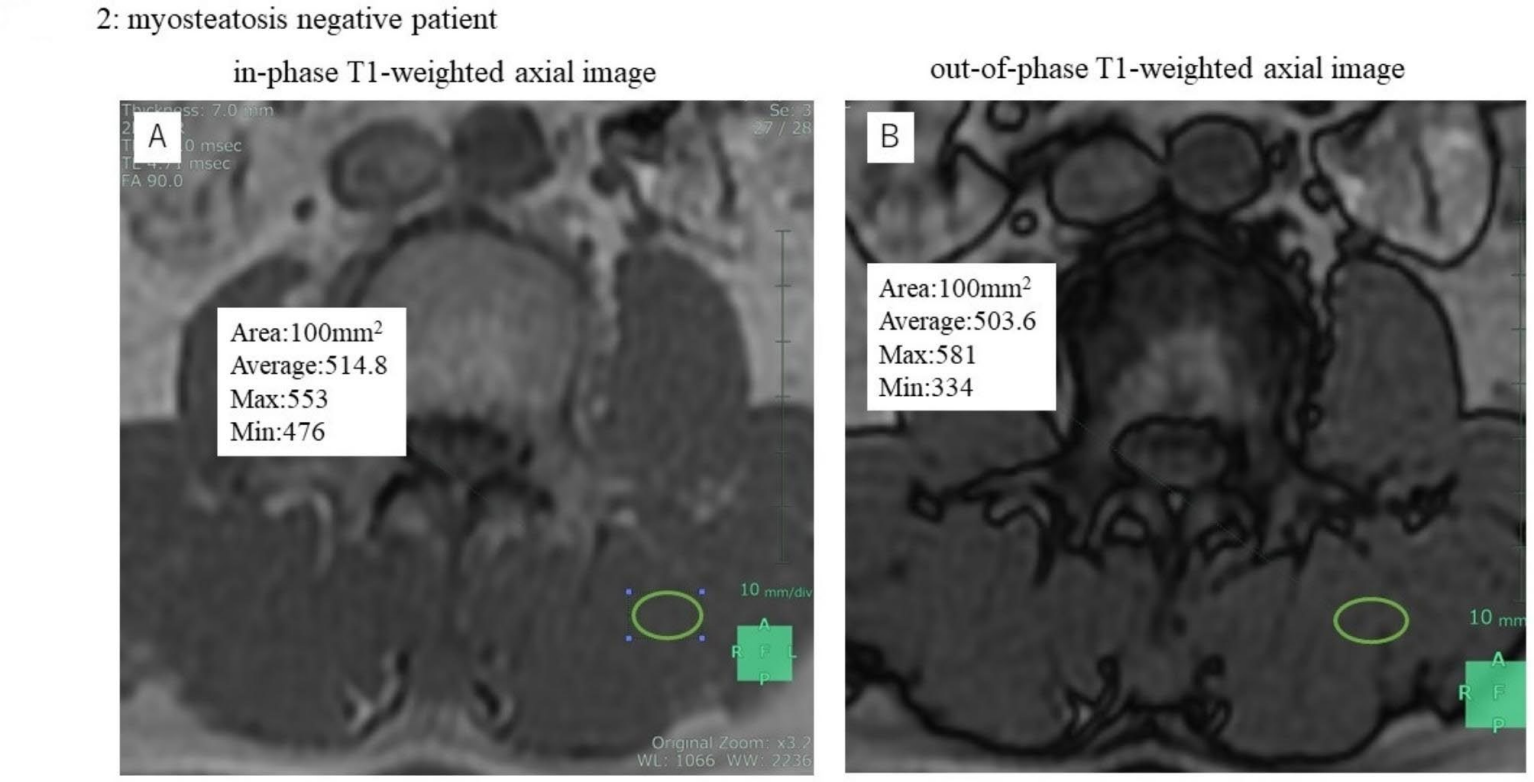
The representative MRI findings in myosteatosis negative patient in-phase T1-weighted axial image out-of-phase T1-weighted axial image

A single 100-mm^2^ oval or round region of interest (ROI) was set in the center of the left multifidus muscles on an in-phase T1-weighted axial image (Fig. 1A A). Each ROI were set manually by the same surgeon at the mid-level of the L3 vertebral body as seen on the coronal MRI scan. The ROI in the in-phase image was copied and pasted onto the out-of-phase image (Figs. 1B and 2B). L3 level is suitable for checking the myosteatosis because L3 is in middle of lumbar vertebra and area around here is largest [20].

The average SI of each ROI was measured. In this study, the SI of the ROI in the in-phase image was defined as SI_*in*_ and the average SI of the ROI in the out-of-phase image was defined as SI_*out*_.

Given that the in-phase MRI scan is acquired when the water and fat are in the same phase, the SI of in-phase is the sum of the water and fat signal intensities:

1 $${SI}_{in}={SI}_{water}+{SI}_{fat}$$

The out-of-phase MRI scan is acquired when the water and fat are in the opposite phases, so the SI of out-of-phase is the difference between the water and fat signal intensities:

2 $${SI}_{out}={SI}_{water}-{SI}_{fat}$$

From formulae (1) and (2), SI_*water*_ and SI_*fat*_ are calculated as follows:

3 $${SI}_{water}= \frac{{SI}_{in}+{SI}_{out}}{2}$$

4 $${SI}_{fat}= \frac{{SI}_{in}- {SI}_{out}}{2}$$

Finally, from formulae (3) and (4), the fat fraction of each ROI is calculated as follows:

$$Fat Fraction \left[\%\right]= \frac{{SI}_{fat}}{{SI}_{water}+{SI}_{fat}}*100=\frac{{SI}_{in}-{SI}_{out}}{2{SI}_{in}}*100$$

### Fat fraction

Myosteatosis was diagnosed based on the fat fraction. Patients was divided the mean value refer to the previous report [14]. Myosteatosis was defined as a mean fat fraction of > 2.60% in men and > 6.02% in women.

### Statistical analysis

Categorical variables were compared using the chi-squared test and continuous variables using the unpaired t-test. Cancer-specific survival, overall survival and disease-free survival curves were generated using the Kaplan–Meier method and differences were compared using the log-rank test. Multivariate analysis was carried out based on the Cox proportional hazard regression model. For all statistical analyses, a p-value of less than 0.05 was considered to indicate statistical significance. All statistical analysis was performed using statistical software (JMP 8.0.1., SAS Campus Drive, Cary, NC).

## Results

### Clinicopathological features

Table 1 summarizes the clinicopathological variables for patients in the myosteatosis-negative group (n = 122) and those in the myosteatosis-positive group (n = 65). The age of the myosteatosis-negative group is 66 ± 0.89 years and myosteatosis-positive group is 73 ± 1.22 years (p < 0.01). The total-bilirubin of the myosteatosis-negative group is 0.86 ± 0.02 mg/dL and myosteatosis-positive group is 0.77 ± 0.03 mg/dL (p = 0.03). The albumin of the myosteatosis-negative group is 4.0 ± 0.04 g/ml and myosteatosis-positive group is 3.8 ± 0.05 g/ml (p = 0.02). There were no significant difference between-two groups in sex, comorbidities, or liver function. The proportion of tumors that were moderately differentiated was significantly higher in the myosteatosis-negative group (p = 0.04). There were no significant difference between-two groups in tumor number, tumor size or stage.

**Table 1:** Patient characteristics

Parameters	Myosteatosis negative (n = 122)	Myosteatosis positive (n = 65)	p value
*Preoperative parameters*			
Age (years)	66 ± 0.89	73 ± 1.22	< 0.01
Sex (male/female)	93/29	47/18	0.55
BMI	23.4 ± 0.29	24.1 ± 0.40	0.16
Etiology (HBV・HCV/Others)	70/52	32/33	0.28
DM (- / +)	78/44	41/24	0.51
HT (- / +)	66/56	25/40	0.07
Plt (×104/µL)	18.2 ± 0.72	19.1 ± 0.99	0.44
T-bil (mg/dL)	0.86 ± 0.02	0.77 ± 0.03	0.03
Alb (g/ml)	4.0 ± 0.04	3.8 ± 0.05	0.02
TLC (/µl)	519 ± 80	700 ± 112	0.19
NLR	2.51 ± 0.13	2.81 ± 0.19	0.20
ICG R15 (%)	11.6 ± 0.85	14.0 ± 1.16	0.10
Child-Pugh class (A/B)	120/2	63/2	0.73
AFP (ng/ml)	3393 ± 1813	5185 ± 2474	0.56
Tumor factor			
Tumor number (Solitary/Multiple)	95/27	53/12	0.55
Tumor size (cm)	4.33 ± 0.26	4.70 ± 0.35	0.40
Diff (Well/Mode/Poor)	17/92/13	11/49/5	0.04
vp (- / +)	101/21	51/14	0.47
vv (- / +)	113/9	64/1	0.06
Stage (I/II/III/IV)	21 / 60 / 34 / 7	8 / 35 / 19 / 3	0.80
Perioperative parameters			
Type of surgery (major/minor)	96/26	54/11	0.46
Operative time (min)	306 ± 6.2	296 ± 8.4	0.37
Blood loss (ml)	235 ± 20	174 ± 27	0.07
Postoperative complication (< Grade III / ≥Grade III)	121/1	65/0	0.35

BMI: body mass index

HBV: hepatitis B Virus

HCV: hepatitis B Virus

DM: diabetes mellitus

HT: hypertension

Plt: platelet count

T-bil: total bilirubin

Alb: albumin

TLC: total lymphocyte count

NLR: neutrophil-to-lymphocyte ratio

ICG: indocyanine green

AFP: α-fetoprotein

Diff: differentiation

vp: portal vein invasion

vv: hepatic vein invasion

### Perioperative features

There was no significant between-group difference in type of surgery performed, operation time. The blood loss of the myosteatosis-negative group is 235 ± 20 ml and tend to be higher than the myosteatosis-positive group is 174 ± 27 ml (p = 0.07). The complication over grade III of two group is quite low and there is no significant difference (myosteatosis-negative group: 1/122, myosteatosis-positive group: 0/65).

### Cancer-specific, overall, and disease-free survival rates

The cancer-specific survival rate after hepatectomy for HCC was significantly worse in the myosteatosis-positive group than in the myosteatosis-negative group (p = 0.03) (Fig. 3A). The 5-year cancer-specific survival rate was 71.7% in the myosteatosis-positive group and 88.4% in the myosteatosis-negative group. Neither the overall survival rate nor the disease-free survival rate (Fig. 3B) was worse in the group with a high fat to tissue ratio (p = 0.17 and p = 0.59, respectively).

**Fig. 3A:**
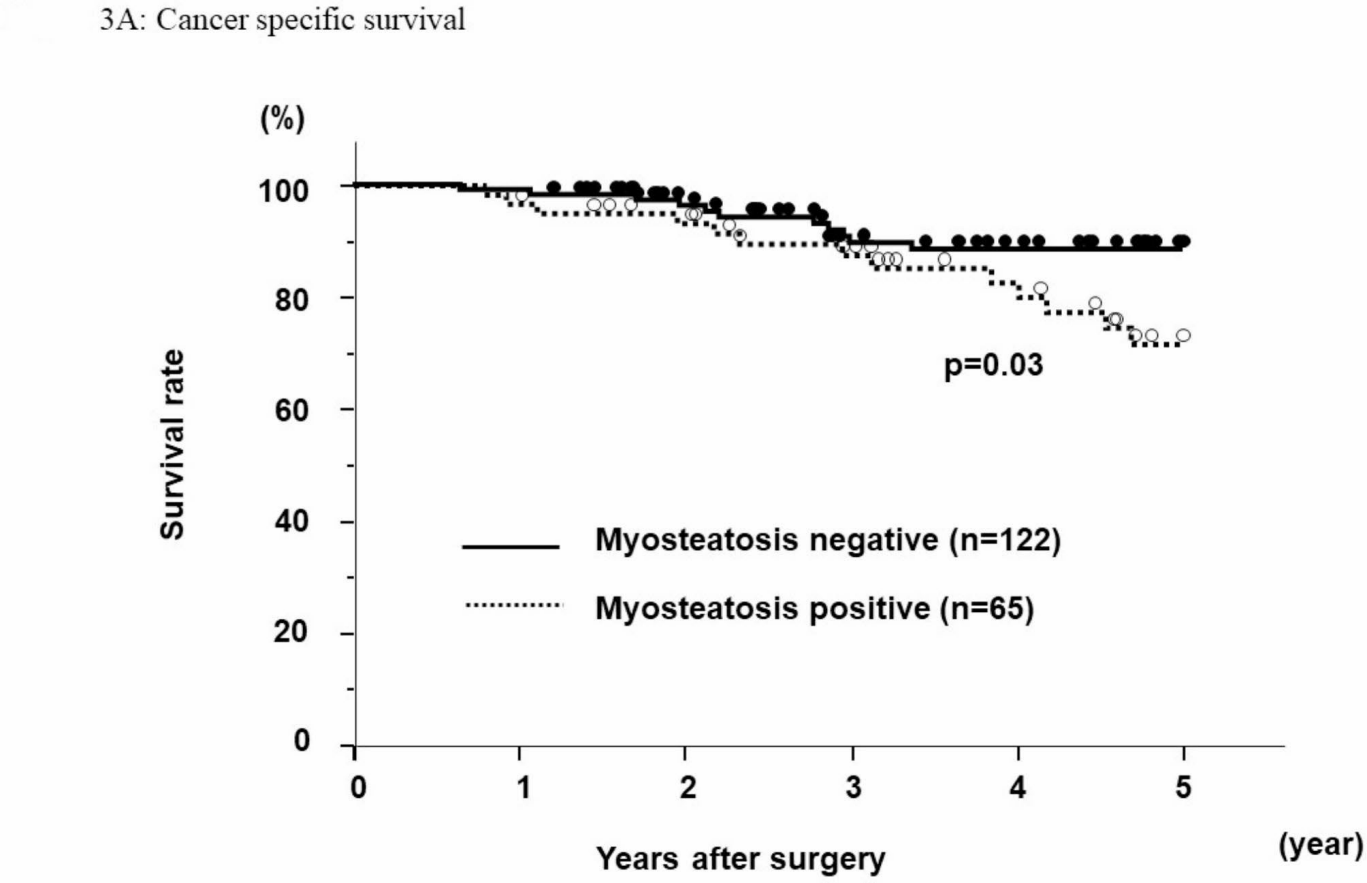
The cancer specific survival Myosteatosis positive group have poor cancer specific survival

**Fig. 3B:**
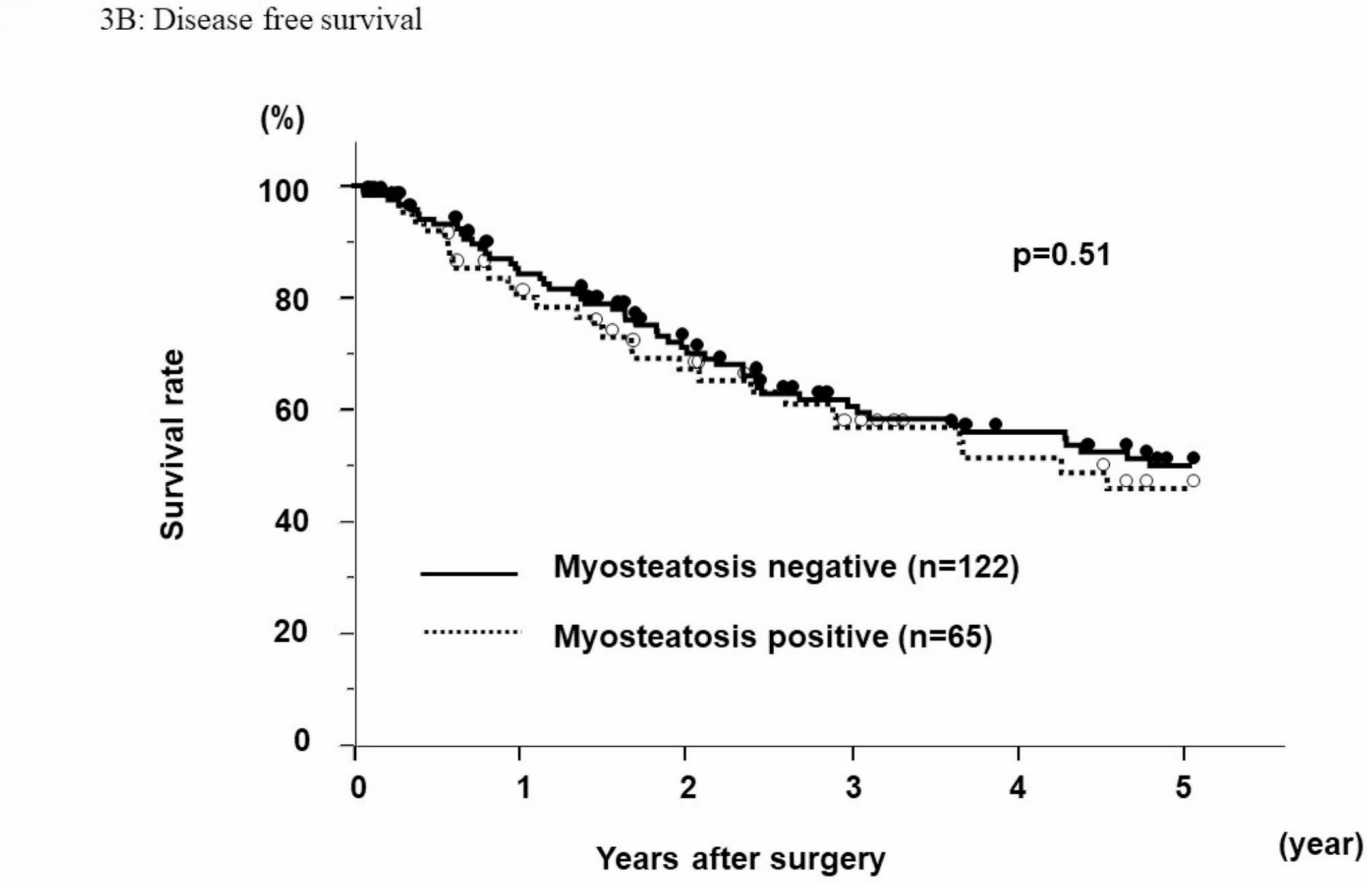
The disease free survival There is no significant difference between two groups

Univariate analysis for cancer specific survival identified multiple tumors (p < 0.01), stage III/IV disease (p < 0.01), an alfa-fetoprotein level ≥ 10 ng/ml (p < 0.01), PIVKA-II ≥ 400 AU/ml (p < 0.01), vp(+) status (p < 0.01), and myosteatosis (p = 0.03) to be prognostic factors for cancer-specific survival. On the multivariate analysis, multiple tumors (hazard ratio 4.30, p < 0.01), alfa-fetoprotein level ≥ 10 ng/ml (hazard ratio 3.51, p = 0.01), myosteatosis (hazard ratio 2.47, p = 0.03) remained as independent predictive factors of cancer specific survival. (Table 2).

**Table 2:** Multivariate analysis for cancer-specific survival

Variable	5 year survival rate(%)	Univariate p Value	Multivariate HR (95% CI)	Multivariate p Value
Age (< 75 / ≥75)	85.4 / 76.4	0.31		
Sex (M/F)	83.4 / 81.6	0.72		
Tumor number (single / multi)	89.0 / 59.0	< 0.01	4.30 (1.50–14.1)	< 0.01
Tumor size (< 2/≥2 cm)	93.2 / 80.1	0.08	2.76 (0.58–20.7)	0.21
ICG R15 (< 15/≥15 cm)	83.8 / 80.3	0.57		
Differentiation (well-mod / por)	84.3 / 69.0	0.13		
Stage (I,II / III, IV)	90.8 / 68.0	< 0.01	1.35 (0.31–6.32)	0.68
AFP (< 10/≥10 ng/ml)	92.8 / 73.8	< 0.01	3.51 (1.25–11.9)	0.01
PIVKA-II (< 400/≥400 AU/ml)	90.5 / 66.9	< 0.01	1.84 (0.74–4.78)	0.18
vp ( - / +)	86.6 / 67.1	< 0.01	1.24 (0.40–3.99)	0.7
vv ( - / +)	84.5 / 48.6	0.07	3.84 (0.76–15.1)	0.09
Myosteatosis (- / +)	88.4 / 71.7	0.03	2.47 (1.04–6.10)	0.03

vp: portal vein invasion

vv: hepatic vein invasion

### Recurrence pattern and treatment

Fifty-two patients had a recurrence and there is no significant difference in recurrence rate between two groups. (myosteatosis positive group 18/65, myosteatosis negative group 34/122 p = 0.97). Furthermore, there is no significant difference in recurrence pattern. (myosteatosis positive group: intrahepatic/extrahepatic 16/2, myosteatosis negative group: intrahepatic/extrahepatic 25/16 p = 0.17). The chemotherapy was performed for 57 patients and there is no significant difference between two groups. (myosteatosis positive group 18/65, myosteatosis negative group 39/122 p = 0.54).

## Discussion

In this study, we investigated the value of myosteatosis as a prognostic marker in patients undergoing radical liver resection for HCC. Our main finding was that myosteatosis is a useful predictor of cancer-specific survival after curative surgery in these patients.

Development of MRI-based methods for assessment of muscle mass and fat content is important considering that MRI are increasingly replacing CT in the diagnostic workup for various cancers. The method of evaluation myosteatosis using CT was established in various cancer. In CT analysis, CT value was used for diagnosis. However, there are few MRI-based studies on myosteatosis in the literature.

Patients with myosteatosis had a poorer transarterial chemoembolization (TACE) response and had a shorter overall survival rate. This study showed the relationship between myosteatosis and surgical treatment [21].

Myosteatosis is defined as increased infiltration of intermuscular and intramuscular fat and is seen on an MRI scan as adipose tissue in between the muscle tissue rather than steatosis of the muscle tissue itself [14]. In addition to loss of muscle mass, aging contributes to redistribution of adipose tissue whereby subcutaneous fat becomes relocated to a less favorable site, such as intramuscular and intermuscular adipose tissue [5, 22] Myosteatosis correlated with fatty infiltration and is associated with metabolic abnormalities and decreased physical strength and mobility. Muscle wasting is a symptom of cancer cachexia, a multifactorial condition that affects the prognosis and a patient’s quality of life negatively [23].

In gastric cancer and breast cancer, myosteatosis is associated with poor prognosis and increase of post-operative complications, furthermore it influences the treatment response in HCC [24].

The mechanisms of myosteatosis and its association with increased risk of tumor relapse and mortality is not dissolved. Three potential mechanisms are proposed. First, myosteatosis is an indicator of a patient’s reduced capacity to withstand the aggressive multimodal therapies required for curative surgical treatment of HCC [3]. Increased intermuscular adipose tissue is associated with physical inactivity and can coincide with reduced cardiorespiratory and overall fitness. Second, myosteatosis is associated with abnormal glucose metabolism, which may facilitate progression of cancer [4, 11]. Finally, myosteatosis has been confirmed to be related to the systemic inflammatory response. The systemic inflammatory response, as calculated by various inflammation-based prognostic scores, is an important indicator of a poor prognosis.

Our data suggest that myosteatosis is an accurate and easy to use predictor of cancer-specific survival in patients with HCC. Therefore, by diagnosis and intervention for myosteatosis, development of sarcopenia is potentially prevented, thereby improving the nutritional status of patients as early as possible in better functional status at the time of surgery [6]. Muscle wasting is a crucial feature of cancer cachexia, which is a multifactorial condition that negatively impacts the prognosis and quality of life [23]. Therefore, it could be a useful predictor of the likelihood of survival when scheduling intervention strategies and tailoring treatment arrangements and may improve survival outcomes in patients with HCC.

In this study, only cancer-specific survival had a significant difference. In general HCC frequently have a recurrence, disease free survival and overall survival are affected by various factors.

As the pre-operative nutritional condition affect the post-operative complication and survival, the evaluation of myosteatosis is very important. In this study, MRI was used for the evaluation of myosteatosis. MRI was performed for the HCC diagnosis, and as this study suggested, myosteatosis was also evaluated using MRI. If the MRI can predict the patient’s nutritional condition before the operation, medical stuff intervene the patient’s condition by excise and nutritional support. And patients have a chance to improve their nutritional condition and improve not only their postoperative complication but also the cancer-specific survival. Previous study showed the usefulness of the perioperative exercise and nutritional support, and these intervene lead to earlier resumption of daily activity [25, 26].

In multivariate analysis, AFP, multi-tumor, and myosteatosis were pointed out as the predictive factors, other studies showed that PIVKA is the predictive factor. Although there is no obvious correlation between myosteatosis and tumor condition directly, many factors influence cancer-specific survival [27].

This study had some limitations. First, it had a single-center study with retrospective design, which could have introduced several sources of bias. Second, we were unable to evaluate the impact of myosteatosis on short-term outcomes because of the low postoperative complication rate. Third, the lack of a standardized definition of myosteatosis. Lastly, there are some other factors influenced the survival.

In conclusion, the findings of this study indicate that myosteatosis measured by MRI is associated with cancer-specific survival after surgery for HCC.

## Data Availability

The datasets used and/or analyzed during the current study are available from the corresponding author on reasonable request.
